# Various Forms of Tuberculosis in Patients with Inflammatory Bowel Diseases Treated with Biological Agents

**DOI:** 10.1155/2021/6284987

**Published:** 2021-01-05

**Authors:** Adam Krusiński, Anna Grzywa-Celińska, Katarzyna Szewczyk, Luiza Grzycka-Kowalczyk, Justyna Emeryk-Maksymiuk, Janusz Milanowski

**Affiliations:** ^1^Chair and Department of Pneumonology, Oncology and Allergology, Medical University of Lublin, Lublin, Poland; ^2^Chair and Department of Pharmaceutical Botany, Medical University of Lublin, Lublin, Poland; ^3^I Department of Medical Radiology, Medical University of Lublin, Lublin, Poland; ^4^Chair of Internal Medicine and Department of Internal Medicine in Nursing, Medical University of Lublin, Lublin, Poland

## Abstract

Although there are undeniable advantages of treatment of the inflammatory bowel diseases, Crohn's disease, and ulcerative colitis, with biological agents, the increased susceptibility to tuberculosis should not be ignored. Tuberculosis is an infectious disease caused by the *Mycobacterium tuberculosis complex* which includes *M. tuberculosis*, *M. bovis*, and *M. africanum*. Primary tuberculosis is uncommon in the setting of inflammatory bowel disease: reactivation of latent tuberculosis is of greater concern. Consequently, latent infection should be excluded in patients who qualify for immunosuppressive treatments. Apart from the review of the literature, this article also presents three cases of different patterns of tuberculosis that occurred during treatment with infliximab, adalimumab, or vedolizumab. The first case reports a case of tuberculosis presenting as right middle lobe pneumonia. The second case featured miliary tuberculosis of the lungs with involvement of the mediastinal lymph nodes, liver, and spleen. The third patient developed a tuberculoma of the right parietal lobe and tuberculous meningitis. It is important to reiterate that every patient qualifying for a biologic agent should undergo testing to accurately identify latent tuberculosis, as well as precise monitoring for the possible development of one of the various forms or patterns of tuberculosis during treatment.

## 1. Introduction

It is well known that treatment with biological agents for various medical conditions for many patients was revolutionary and provided a real chance for positive shift in the course and prognosis of the underlying disease. Biotherapies have become applicable not only in the treatment of inflammatory bowel diseases (IBD), Crohn's disease (CD), and ulcerative colitis (UC) but also in the treatment of such conditions as rheumatoid arthritis (RA), psoriatic arthritis (PsA), and ankylosing spondylitis (AS) [1] and in therapy of dermatological diseases such as plaque psoriasis [2] and hidradenitis suppurativa (HS) [3]. These biotherapies have also been evaluated in pulmonary diseases such as asthma, but, despite promising results from preclinical studies, they have proved to be ineffective [4].

In spite of the unquestionable benefits of these biotherapies, particularly in difficult-to-treat cases of IBD, it is important to not overlook the fact that, in some cases, biological treatments may lead to serious adverse reactions. One example is the reactivation of latent infection with *Mycobacterium tuberculosis* or new-onset tuberculosis (TB).

Although both CD and UC share features of uncontrolled and relapsing inflammation, they can differ in terms of clinical features, etiology, and treatment. In 5% to 15% of cases (more often among children), it is not possible to differentiate based on the endoscopic or histological examination; in such situations, the term *inflammatory bowel disease unclassified* (IBDU) is used to describe the condition [5].

CD is an inflammatory, autoimmune-related disease of unclear etiology, which may involve each part of the gastrointestinal tract, especially the small intestine. The disease is characterized by full-thickness, segmental changes with the presence of noncaseating granulomas; it can be complicated by the development of abscesses, fistulae, or perianal changes. In patients with CD, parenteral symptoms are often observed (affecting the skin, choroid, joints, liver, and bile ducts). Moreover, patients have a higher risk of developing colorectal cancer [6].

The first-line agents in the treatment of CD are often corticosteroids in combination, in case of extensive involvement of the small intestine, with steroid-sparing immunosuppressive medications such as azathioprine, mercaptopurine, and methotrexate. In case of infection or the presence of fistulae, antibiotics such as ciprofloxacin and metronidazole and, subsequently, biological agents are also used [6].

UC is characterized by continuous inflammatory changes typically extending from the rectum, with involvement limited to the large bowel. In contrast to CD, in UC, the inflammation is limited to the mucosa.

In UC, the drugs such as 5-aminosalicylic acid, budesonide, and beclomethasone are used. In patients who have required, at least, two courses of corticosteroid therapy in the preceding 12 months, the *British Society of Gastroenterology* recommends the escalation of the treatment by using a thiopurine, antitumour necrosis factor (TNF) therapy, vedolizumab, or tofacitinib [5].

## 2. Biological Treatment of IBD

In the case reports described in the later part of this article, adalimumab, infliximab, and vedolizumab were used. The first two agents belong to the group of TNF*α* inhibitors with the structure of IgG_1._ TNF*α* is a cytokine that plays an essential role in the pathogenesis of several inflammatory disorders; it is secreted by macrophages and T cells and has strong proinflammatory effects. It also plays a relevant role in the immune responses against microorganisms and neoplastic cells. Its main action, among others, is activation of pathways leading to apoptosis and cell necrosis [7]. Increased TNF*α* concentrations are seen in several autoimmune diseases [8].

Infliximab—a chimeric human-mouse antibody with high affinity for human TNF*α*—was first launched in 1998 and was the first biological agent approved for the treatment of moderate-to-severe CD and UC. Studies have demonstrated efficacy of infliximab for the induction of remission and maintenance in patients, including those with complicated disease (such as fistulising disease) [9, 10]. Apart from IBD, infliximab is also indicated for ankylosing spondylitis, psoriasis, and psoriatic arthritis [10]. The results of long-term prospective studies by Lichtenstein et al. [11] showed that therapy with infliximab involves a similar risk of death as in case of classical medicinal products; however, infliximab was associated with a more frequent occurrence of serious infections and autoimmune and demyelinating diseases.

Adalimumab—a recombinant human antibody against TNF*α*—is indicated for use in moderate-to-severe active rheumatoid arthritis when previously administrated therapy with immunosuppressants, glucocorticosteroids, or infliximab was poorly tolerated or inefficient. Additionally, adalimumab induces apoptosis in human monocytes [12]. Early commencement of a biotherapy slows down the progression of the disease [13] and allows the avoidance of polytherapy [14].

Therapy with adalimumab is considered to be relatively safe [15]. The results of the study by Tanaka et al. [16] demonstrated that four years after starting adalimumab treatment, therapy was continued in 62% of patients. However, Lehtola et al. [17] in a 2-year observation of 100 patients with nonspecific IBD noted that just 29 remained in remission. Sixty-three patients discontinued the therapy, and 36 patients with CD underwent a surgery procedure to manage symptoms of the underlying condition [17]. Adalimumab is highly effective in treating fistulising CD, and its effectiveness in closing gaps has been shown in both adults and children [18–20]. The agent can be also used in maintenance treatment to sustain remission. Before initiating treatment with adalimumab, the presence of TB and opportunistic infections (especially *P. jiroveci*, but also *Hepatitis B* and *C* viruses should be taken into account) must be excluded [21]. The authors of another study indicated efficacy of adalimumab in patients with small intestine strictures [22]. In the multicentre study, CREOLI Buhnik et al. demonstrated that 64% of patients with symptomatic small bowel stricture (SSBS) did not have to undergo additional therapeutic interventions while using adalimumab [22]. Due to increased risk of lung and head/neck cancers, caution should be exercised in smokers and patients with COPD [5].

Vedolizumab (marketed in the EU and USA since 2014) is a new agent indicated for use in IBD. Vedolizumab is a novel therapeutic monoclonal antibody acting selectively in the gut *via* binding to the *α*4*β*7 integrin present on activated B and T cells. This protein is a receptor binding the mucosal addressin cell adhesion molecule 1 (MAdCAM1), and its blocking inhibits migration of lymphocytes into the gut, thus reducing local inflammations [23, 24]. This mode of action does not result in systemic immunosuppression and, consequently, should not increase the risk of cancer or opportunistic infections, including TB. Those findings were confirmed by Ng et al. [25] where TB among study participants was observed rarely and reactivation of HBV and HCV infections was not seen [26]. Results of the subsequent study by Colombel et al. [27] involving 2,830 patients with nonspecific IBD demonstrated occurrence of TB, sepsis, and *Clostridium* infections in up to 0.6% patients. Results from numerous studies indicate that vedolizumab is efficient in inducing and sustaining remission and is considered to be safe and well tolerated [23, 24, 26, 28]. Studies involving patients with UC suggest that vedolizumab is effective, especially as a second-line treatment after previous therapy with TNF*α* inhibitors [28, 29]. The results of the study of Reenaers et al. [30] demonstrate its superior efficacy as a first-line biological treatment in patients with moderate-to-severe IBD. Despite this, it is still recommended to not use vedolizumab in patients with active TB and to detect and treat latent TB in each patient before initiating vedolizumab [31].

## 3. Biological Treatment and Tuberculosis

Tuberculosis (TB) is an infectious disease caused by the *Mycobacterium tuberculosis complex* which includes *M. tuberculosis*, *M. bovis*, and *M. africanum*. In the initial stage, *M. tuberculosis* cells are phagocytized by macrophages. They rapidly multiply inside the dead macrophages, and after disintegration of macrophages, mycobacteria form granulation tissue composed of granular caseation necrosis and attack the successive cells. At this point, activation of T cells and intensification of cellular responses are observed. Initially, the infection may be asymptomatic; however, TB bacteria can remain latent for many years and then, in favourable conditions, become active. Therefore, latent (LTBI), as well as an active tuberculosis, infection should be excluded in patients who qualify for immunosuppressive treatments, especially those with anti-TNF*α* agents [32].

Due to the airborne route of infection, the lung is the predominant site of TB. The clinical presentation is nonspecific. Typically, a chronic cough and, less often, haemoptysis or dyspnoea are observed. On physical examination, especially in the initial stages of the disease, auscultatory changes may be absent. General symptoms of TB include low-grade fever, hyperhidrosis, decreased appetite, and weight loss. However, it should be noted that the tuberculous process can affect any organ of the body, especially when it comes to hematogenous spread [33].

TB is an uncommon complication of treatment with TNF*α* inhibitors; however, studies in patients with rheumatic diseases revealed increased risk for TB in patients with biotherapies. In these studies, 0.21% of patients treated with infliximab, 0.2% treated with adalimumab, and 0.05% treated with etanercept developed tuberculosis during the course of therapy [33].

Tests for the diagnosis of pulmonary tuberculosis disease include a chest X-ray examination and the gamma interferon (IFN-*γ*) release assay (IGRA), which provides an alternative to a routine tuberculin test (of a lower diagnostic value, especially in patients previously vaccinated with BCG) [34]. It should be noted that false-negative IGRA test results may occur in patients with impaired cell-mediated immune responses. Detecting the presence of the bacteria, especially in a bacterial culture testing, is the conclusive method of TB diagnosis. However it is possible to diagnose TB without positive bacteria culture test results [35]. The sequencing of the entire *Mycobacterium* genome also appears to be a promising method of TB detection [36].

A typical TB treatment regimen includes two months of rifampicin, isoniazid, ethambutol, and pyrazinamide and then a further four months of rifampicin and isoniazid only. Tuberculosis treatment should be prolonged to, at least, nine months in patients with underlying immunodeficiency or those receiving an immunosuppressive therapy. In the setting of TB induced by a TNF-*α* inhibitor, this agent should be discontinued, although this may not always be necessary [36]. There is no consensus on whether it is safe to readminister biological treatment in patients with IBD who have a disease exacerbation after withdrawal of a biologic therapy due to active tuberculosis. Similarly, there are no guidelines defining the optimal time for the reintroduction of biological treatment in patients who have started antituberculosis treatment.

The data in the literature are sparse and refer mainly to patients with rheumatic diseases. In one paper describing the readministration of TNF*α* inhibitors in patients with RA or AS who developed active tuberculosis whilst on anti-TNF*α* therapy, the median duration from cessation of anti-TNF*α* therapy to reintroduction was 3 (range 2–7) months in RA and 12 (range 6–29) months in AS [37].

In another study involving 21 patients (two of whom had CD) who developed TB during TNF*α* blocker treatment, six patients recommenced TNF*α* blockers at 2 (*n* = 1), 3 (*n* = 1), 7.5 (*n* = 1), and 12 months (*n* = 3) after the initiation of anti-TB treatment [38].

In another paper describing 13 patients with rheumatic disease who developed active TB infection during treatment with a TNF*α* inhibitor, the TNF*α* inhibitor treatment was reinitiated in six patients: four within 2 months after TB treatment and two after completion of TB treatment [39].

There are opinions that the biological treatment may be reinitiated after one month of adequate anti-TB therapy (where the susceptibility of the tubercle bacilli to anti-TB agents is shown) [35], but we believe that the biological treatment should be interrupted for, at least, three months if possible.

Preventative TB treatment in patients qualified to receive TNF*α* inhibitors is recommended in case of positive tuberculin skin or IRGA test results (current or historical), history of ineffectively treated TB, or contact with an individual with active TB disease [35]. The treatment includes isoniazid monotherapy or in combination with rifampicin or rifapentine, or possibly rifampicin in monotherapy. Use of isoniazid in combination with rifapentine allows shortening therapy to three months, with an efficiency of 60–90% [40]. However, TB development is possible despite standard chemoprophylaxis [41, 42].

Since TB usually develops as reactivation of latent infection in adults, it is crucial that the host immune system is able to control the *M. tuberculosis* population. Cell-mediated immune response based on CD4+ lymphocytes and cytokines (*i.e.,* IFN*γ*, TNF*α*, and IL-12) plays a key role. In the course of TB, infected dendric cells (DCs) migrate to lymph nodes where mediated by IL-12 activate T cells into the Th1 phenotype. Those lymphocytes, after returning to the lungs, secrete IFN*γ* which stimulates infected macrophages to produce TNF*α* (however, it is also secreted by neutrophils, DCs, and lymphocytes themselves). TNF*α* has pleiotropic properties associated with cellular response, *i.e,* when activating macrophages and CD4+ lymphocytes and inducing production of other proinflammatory cytokines, including IFN*γ*. It seems that, in the course of TB, TNF*α* plays a vital role in forming and maintaining granulomas. It is suggested that granulomas may be a form of infection control keeping bacteria in one place. Moreover, TNF*α* accelerates intracellular elimination of mycobacteria; its blocking inhibits phagosomal maturation [43]. Another role of TNF*α* is induction of apoptosis of infected cells *via* activation of the caspase cascade. Use of TNF*α* inhibitors may also cause immunosuppression as a result of intensification of Treg cell responses, which have anti-inflammatory effects [44].

Tests on mice with blocked TNF*α* indicated that the animals were very susceptible to *M. tuberculosis* infection, and latent infections were reactivated. As noted, it happened with unchanged responses associated with IFN*γ* and IL-12. It is suggested that TNF*α* plays a special role in the control of latent infection. Studies on humans revealed a five-fold increase in the incidence of TB with suppressed TNF*α*, whereby 25% of patients had miliary tuberculosis and 33% of patients had single extrapulmonary foci, which suggested reactivation of latent infection [44, 45].

It has been shown that anti-TNF biological treatments are associated with increased risk for TB [46] and risk of contracting the disease is higher for anti-TNF*α* monoclonal antibodies than with soluble TNF*α* receptor therapy [47].

In view of delayed clearance of biological agents after cessation, patients receiving biological therapies should be monitored for TB for a period of five months after discontinuation of adalimumab therapy and for six months after the end of infliximab treatment [5, 48].

## 4. Three Forms of Tuberculosis Developed during the Treatment of IBD with Biological Agents

In our clinical practice, as biological treatments are increasingly used, we have noted several cases of TB that developed during treatment with a biological therapy. Below, we briefly present cases of three patients with IBD in whom TB developed soon after initiating treatment with a biological agent. Each of those cases is different; two of those had a dramatic course. Therefore, the aim of this report is to highlight that various types of TB disease should be considered at the point of planning to use a biological treatment not only in patients with IBD but also in other areas of medicine.

**Case 1 .**

**Figure 1:**
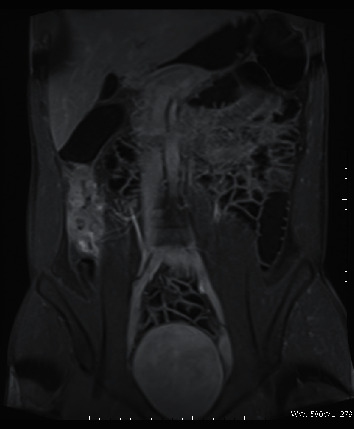
Coronal t1-weighted MRI image with the gadolinium contrast agent, presenting wall thickening and enhancement of the caecum and proximal ascending colon.

**Figure 2:**
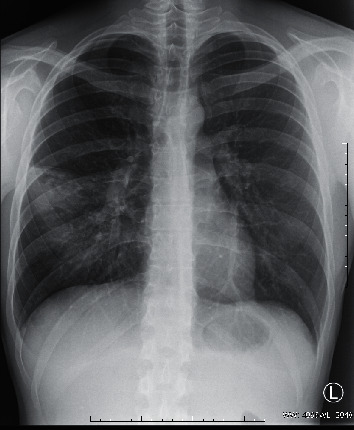
Diffuse consolidation in the lower lobe of the right lung (segment 6) consistent with pneumonia.

A 25-year-old patient with CD (Figure 1) treated with adalimumab and azathioprine for several months was admitted to hospital due to fever of 40°C that lasted for ten days. Before hospitalization, the patient had been ineffectively treated with cefuroxime. We noted high inflammatory laboratory parameters, a positive IGRA test result, and negative blood culture results. A sputum sample for a culture testing was not obtained. X-ray examination showed features of inflammation of the right middle lobe (RML) (Figure 2). The patient received empirical treatment with ceftazidime, amoxicillin with clavulonic acid, clarithromycin, and acyclovir. *M. tuberculosis* infection was subsequently confirmed by molecular testing, culture tests, and bacterioscopic examination of bronchial aspirate. After commencing the antimycobacterial treatment, rapid clinical and laboratory improvements were observed. He was maintained on mesalazine and a probiotic for his CD, without worsening. The patient was discharged from hospital and transferred to a tuberculosis sanatorium for further treatment.

**Case 2 .**

**Figure 3:**
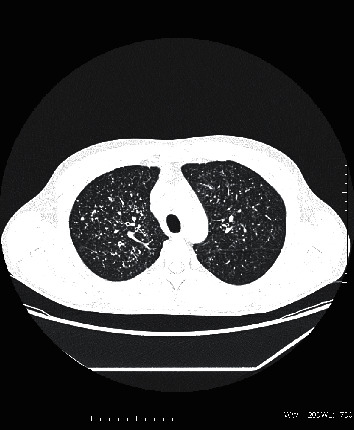
Axial chest computed tomography with the presence of innumerable small (2–4 mm) pulmonary nodules with a centrilobular predilection, consistent with miliary tuberculosis.

**Figure 4:**
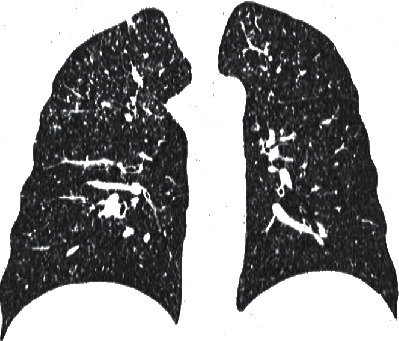
Coronal plane chest CT with the presence of innumerable small (2–4 mm) pulmonary nodules with a centrilobular predilection, consistent with miliary tuberculosis.

**Figure 5:**
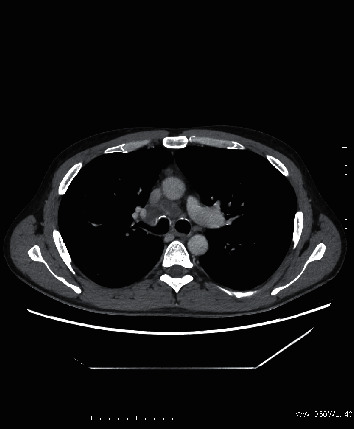
Axial contrast-enhanced CT scan with the presence of enlarged, necrotic mediastinal lymph nodes at the level of carina.

A 37-year-old patient with CD was initially diagnosed as pseudomembranous colitis complicated by perianal fistulae and abscess formations. Right hemicolectomy with partial sigmoid colon resection had been performed in the past. The patient was treated with infliximab for one year. Admission to our clinic was based on the symptoms presented by the patient (dysponea and cough) and the CT results, which indicated the presence of miliary tuberculosis of the lungs (Figures 3 and 4) with mediastinal lymph nodes (Figure 5), hepatic, and splenic involvement. Due to the presence of neurological and mental disorders (agitation and positive psychotic symptoms), a CT of the brain was performed and a sample of cerebrospinal fluid was collected: *M. tuberculosis* was detected with use of a molecular testing (bacteria culture testing- negative; bacterioscopic examination- negative). The sputum culture for *M. tuberculosis* and IGRA test results were positive.

Due to laboratory features of bone marrow *aplasia, M. tuberculosis* spread to the bone marrow was suspected. Treatment included filgrastim, packed red blood cells, platelet concentrate, and fresh frozen plasma. Clinical and laboratory improvements were achieved after initiation of antimycobacterial treatment (amikacin, isoniazid, rifampicin, pyrazinamide, and ethambutol). Management of the patient's CD included mesalazine and a probiotic. The patient was transferred to a sanatorium for further treatment.

**Case 3 .**

**Figure 6:**
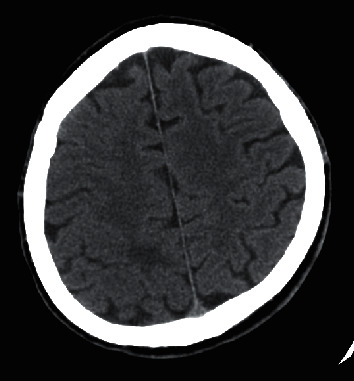
Axial CT image of the brain with a hypodense lesion in the right parietal lobe, surrounded with oedema.

**Figure 7:**
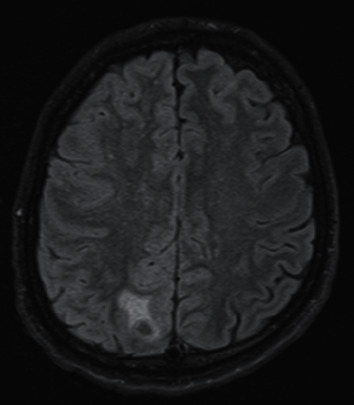
MRI flair image at the same level depicts oedema surrounding a small, nodal lesion.

This 41-year-old patient with UC was treated with vedolizumab. He was hospitalized due to recurrent pleural effusion and managed initially in the Department of Thoracic Surgery. After videothoraoscopy, left hemiparesis and neurological symptoms (suggesting stroke occurrence or epileptic seizure) were observed. Based on histopathological examination of pleural fluid, tuberculous pleuritis was diagnosed. The MRI of the brain revealed the presence of tuberculoma of the right parietal lobe (Figures 6 and 7) and tuberculous meningitis. Due to deteriorating respiratory failure, the patient was transferred to the Intensive Care Department were TB was confirmed based on the results of bronchial aspirate culture. Results of the IGRA test were indeterminate. The patient was transferred to our clinic where treatment included management of oedema (dexamethasone, mannitol, and furosemide), sedative (benzodiazepine, haloperidol, and quetiapine), and antimycobacterial agents (amikacin, isoniazid, rifampicin, pyrazinamide, and ethambutol). His UC treatment included mesalazine and hydrocortisone. The neurological and mental symptoms continued despite regression of the lesions noted on repeat MRI of the head. The patient was transferred to a sanatorium for further treatment.

## 5. Is It Possible to Reduce the Risk of Developing Tuberculosis in Patients with Inflammatory Bowel Diseases Treated with Biological Agents?

It is well known that the risk of developing TB consequent to latent infection in patients with IBD undergoing biological treatment is increased: first of all, because of the disease itself and, secondly, because of treatment. Tuberculosis can present in different locations: not only as pulmonary disease but also up to 91% can have, at least, one extrapulmonary location [49]. Carpio et al. [50] reported 34% of disseminated tuberculosis and 26% of extrapulmonary localization in the population of 50 TB cases in patients with IBD-treated anti-TNF. These findings, as well as our reports, should lead to the conclusion that different forms of tuberculosis can occur in patients with IBD.

The interval between the beginning of treatment and symptoms or diagnosis of tuberculosis varied in different studies from a median of 6 [50–52] to 14.5 months [49]. Consequently, it is clear that the period of observation should not cover only the start of treatment with biological agents.

Unfortunately, even negative initial screening does not exclude the risk of TB development in these patients [49]. The methods used in screening for TB (e.g., anamnesis, chest X-ray, tuberculin skin test, and IGRA) can be unreliable [49]. The IGRA test seems to be more sensitive than skin testing, but it should be noted that immunosuppression can also lead to false-negative results [52]. To minimalize the risk of not detecting the development of TB in patients treated with biological agents, we recommend annual screening with the IGRA test and a chest X-ray, along with a detailed assessment for TB symptoms. If suspicious symptoms are noted, a full diagnostic workup for possible TB should be performed.

It is always better to prevent than to treat. Patients with IBD receiving a biological treatment should probably follow the WHO recommendations on TB infection prevention [53] more closely than healthy people. These recommendations contain administrative and environmental controls and respiratory protection manners that can reduce the risk of TB transmission in the population. The role of triage and sick patient separation systems, effective treatment of those who have already developed TB, and rigorous respiratory hygiene (e.g., cough etiquette) are emphasized. Another way of lowering the risk of TB transmission mentioned in WHO recommendations is cleaning the air by using high-efficiency particulate air (HEPA) filtration or germicidal ultraviolet systems, especially in populations with high TB occurrence [53]. As practicing clinicians, we should inform and encourage all patients to adhere to these recommendations.

## 6. Conclusions

Preparing patients with CD to receive biological treatments requires accurate identification of latent tuberculosis infections, although this may be difficult due to the effect of the disease itself on the results of diagnostic testing, e.g., IGRA test. Additionally, we should always check for symptoms of the disease, especially as it may be characterized by an atypical course and affect each body organ and system. Negligence in this regard may not only have negative impacts on patients but also have population consequences associated with spreading the infection.

## References

[1] Grabarek B. O., Wcisło‐Dziadecka D., Bednarek K., Kruszniewska‐Rajs C., Gola J. (2019). Assessment of transcriptional activity genes associated with the IL-17 signaling pathway in skin fibroblasts under the influence of adalimumab. *Dermatologic Therapy*.

[2] Wu J. J., Valdecantos W. C. (2017). Adalimumab in chronic plaque psoriasis: a clinical guide. *Journal of Drugs in Dermatology*.

[3] Maarouf M., Clark A. K., Lee D. E., Shi V. Y. (2018). Targeted treatments for hidradenitis suppurativa: a review of the current literature and ongoing clinical trials. *Journal of Dermatological Treatment*.

[4] Mukhopadhyay S., Hoidal J. R., Mukherjee T. K. (2006). Role of TNF*α* in pulmonary pathophysiology. *Respiratory Research*.

[5] Lamb C. A., Kennedy N. A., Raine T. (2019). British society of gastroenterology consensus guidelines on the management of inflammatory bowel disease in adults. *Gut*.

[6] Veauthier B., Hornecker J. R. (2018). Crohn’s disease: diagnosis and management. *American Family Physician*.

[7] Idriss H. T., Naismith J. H. (2000). TNF alpha and the TNF receptor superfamily: structure-function relationship(s). *Microscopy Research and Technique*.

[8] Palladino M. A., Bahjat F. R., Theodorakis E. A., Moldawer L. L. (2003). Anti-TNF-*α* therapies: the next generation. *Nature Reviews Drug Discovery*.

[9] Hemperly A., Vande Casteele N. (2018). Clinical pharmacokinetics and pharmacodynamics of Infliximab in the treatment of inflammatory bowel disease. *Clinical Pharmacokinetics*.

[10] Sands B. E., Anderson F. H., Bernstein C. N. (2004). Infliximab maintenance therapy for fistulizing Crohn’s disease. *New England Journal of Medicine*.

[11] Lichtenstein G. R., Feagan B. G., Cohen R. D. (2018). Infliximab for Crohn’s disease: more than 13 years of real-world experience. *Inflammatory Bowel Diseases*.

[12] Shen C., Assche G. V., Colpaert S. (2005). Adalimumab induces apoptosis of human monocytes: a comparative study with infliximab and etanercept. *Alimentary Pharmacology and Therapeutics*.

[13] Panchal H., Wagner M., Chatterji M. (2019). Earlier anti-tumor necrosis factor therapy of Crohn’s disease correlates with slower progression of bowel damage. *Digestive Diseases and Sciences*.

[14] Colombel J.-F., Jharap B., Sandborn W. J. (2017). Effects of concomitant immunomodulators on the pharmacokinetics, efficacy and safety of adalimumab in patients with Crohn’s disease or ulcerative colitis who had failed conventional therapy. *Alimentary Pharmacology & Therapeutics*.

[15] Colombel J.-F., Sandborn W. J., Reinisch W. (2018). Long-term safety of adalimumab in clinical trials in adult patients with Crohn’s disease or ulcerative colitis. *Alimentary Pharmacology & Therapeutics*.

[16] Tanaka H., Kamata N., Yamada A. (2018). Long-term retention of adalimumab treatment and associated prognostic factors for 1189 patients with Crohn’s disease. *Journal of Gastroenterology and Hepatology*.

[17] Lehtola E., Haapamäki J., Färkkilä M. A. (2016). Outcome of inflammatory bowel disease patients treated with TNF-*α* inhibitors: two-year follow-up. *Scandinavian Journal of Gastroenterology*.

[18] Banasiewicz T., Eder P., Rydzewska G. (2019). Statement of the expert group on the current practice and prospects for the treatment of complex perirectal fistulas in the course of Crohn’s disase. *Polish Journal of Surgery*.

[19] Panaccione R., Colombel J.-F., Sandborn W. J. (2013). Adalimumab maintains remission of Crohn’s disease after up to 4 years of treatment: data from CHARM and ADHERE. *Alimentary Pharmacology & Therapeutics*.

[20] Ruemmele F. M., Rosh J., Faubion W. A. (2018). Efficacy of Adalimumab for treatment of perianal fistula in children with moderately to severely active Crohn’s disease: results from IMAgINE 1 and IMAgINE 2. *Journal of Crohn’s and Colitis*.

[21] Murdaca G., Spanò F., Contatore M. (2015). Infection risk associated with anti-TNF-*α* agents: a review. *Expert Opinion on Drug Safety*.

[22] Bouhnik Y., Carbonnel F., Laharie D. (2018). Efficacy of adalimumab in patients with Crohn’s disease and symptomatic small bowel stricture: a multicentre, prospective, observational cohort (CREOLE) study. *Gut*.

[23] Tarabar D., Hirsch A., Rubin D. T. (2016). Vedolizumab in the treatment of Crohn’s disease. *Expert Review of Gastroenterology & Hepatology*.

[24] Bach L. E., Taleban S. (2020). Vedolizumab (VDZ) for UC and CD: still safe and effective after all these years. *Digestive Diseases and Sciences*.

[25] Ng S. C., Hilmi I. N., Blake A. (2018). Low frequency of opportunistic infections in patients receiving Vedolizumab in clinical trials and post-marketing setting. *Inflammatory Bowel Diseases*.

[26] Meserve J., Dulai P. (2020). Predicting response to Vedolizumab in inflammatory bowel disease. *Frontiers in Medicine*.

[27] Colombel J.-F., Sands B. E., Rutgeerts P. (2017). The safety of vedolizumab for ulcerative colitis and Crohn’s disease. *Gut*.

[28] Singh S., Fumery M., Sandborn W. J., Murad M. H. (2018). Systematic review with network meta-analysis: first- and second-line pharmacotherapy for moderate-severe ulcerative colitis. *Alimentary Pharmacology & Therapeutics*.

[29] Takatsu N., Hisabe T., Higashi D., Ueki T., Matsui T. (2020). Vedolizumab in the treatment of ulcerative colitis: an evidence-based review of safety, efficacy, and place of therapy. *Core Evidence*.

[30] Reenaers C., Cremer A., Dewit O. (2020). Effectiveness and persistence of Vedolizumab in patients with inflammatory bowel disease: results from the Belgian REal-LIfe study with Vedolizumab (Be-RELIVE). *Acta Gastro-Enterologica Belgic*.

[31] Summary of product characteristics: ENTYVIO, https://www.ema.europa.eu/en/medicines/human/EPAR/entyvio

[32] Bulterys M. A., Wagner B., Redard-Jacot M. (2020). Point-of-care urine LAM tests of tuberculosis diagnosis: a status update. *Journal of Clinical Medicine*.

[33] Cantini F., Niccoli L., Goletti D. (2014). Adalimumab, etanercept, infliximab, and the risk of tuberculosis: data from clinical trials, national registries, and postmarketing surveillance. *The Journal of Rheumatology Supplement*.

[34] Dobler C. C. (2016). Biologic agents and tuberculosis. *Microbiology Spectrum*.

[35] Korzeniewska-Koseła M. (2016). Tuberculosis: actual problems with diagnosis and treatment. *Wiadomości lekarskie*.

[36] Mejza F., Niżankowska-Mogilnicka E. (2017). Choroby układu oddechowego—postępy 2016. *Medycyna Praktyczna*.

[37] Suh Y. S., Kwok S.-K., Ju J. H., Park K.-S., Park S.-H., Yoon C.-H. (2014). Safe re-administration of tumor necrosis factor-alpha (TNF*α*) inhibitors in patients with rheumatoid arthritis or ankylosing spondylitis who developed active tuberculosis on previous anti-TNF*α* therapy. *Journal of Korean Medical Science*.

[38] Denis B., Lefort A., Flipo R. M. (2008). Long-term follow-up of patients with tuberculosis as a complication of tumour necrosis factor (TNF)-alpha antagonist therapy: safe re-initiation of TNF-alpha blockers after appropriate anti-tuberculous treatment. *Clinical Microbiology and Infection*.

[39] Kim Y. J., Kim Y.-G., Shim T. S. (2014). Safety of resuming tumour necrosis factor inhibitors in patients who developed tuberculosis as a complication of previous TNF inhibitors. *Rheumatology*.

[40] Mejza F. (2016). Rozpoznanie i leczenie utajonego zakażenia prątkiem gruźlicy: podsumowanie wytycznych Światowej Organizacji Zdrowia 2015. *Medycyna Praktyczna*.

[41] Agudo B., Díaz G., González-Lama Y. (2016). Adalimumab-receiving ulcerative colitis patient suffered latent tuberculosis reactivation despite correct chemoprophylaxis and was successfully treated while on anti-tumour necrosis factor therapy. *Journal of Crohn’s and Colitis*.

[42] Ramos G. P., Stroh G., Al-Bawardy B., Faubion W. A., Papadakis K. A., Escalante P. (2018). Outcomes of treatment for latent tuberculosis infection in patients with inflammatory bowel disease receiving biologic therapy. *Inflammatory Bowel Diseases*.

[43] Harris J., Hope J. C., Keane J. (2008). Tumor necrosis factor blockers influence macrophage responses to *Mycobacterium tuberculosis*. *The Journal of Infectious Diseases*.

[44] Harris J., Keane J. (2010). How tumour necrosis factor blockers interfere with tuberculosis immunity. *Clinical & Experimental Immunology*.

[45] O’Garra A., Redford P. S., McNab F. W., Bloom C. I., Wilkinson R. J., Berry M. P. R. (2013). The immune response in tuberculosis. *Annual Review of Immunology*.

[46] Brassard P., Kezouh A., Suissa S. (2006). Antirheumatic drugs and the risk of tuberculosis. *Clinical Infectious Diseases*.

[47] Tubach F., Salmon D., Ravaud P. (2009). Risk of tuberculosis is higher with anti-tumor necrosis factor monoclonal antibody therapy than with soluble tumor necrosis factor receptor therapy: the three-year prospective French research axed on tolerance of biotherapies registry. *Arthritis & Rheumatism*.

[48] Summary of Product Characteristics: Remicade, https://www.ema.europa.eu/en/search/search?search_api_views_fulltext=Remicade

[49] Abitbol Y., Laharie D., Cosnes J. (2016). Negative screening does not rule out the risk of tuberculosis in patients with inflammatory bowel disease undergoing anti-TNF treatment: a descriptive study on the GETAID cohort. *Journal of Crohn’s and Colitis*.

[50] Carpio D., Jauregui-Amezaga A., de Francisco R. (2016). Tuberculosis in anti-tumour necrosis factor-treated inflammatory bowel disease patients after the implementation of preventive measures: compliance with recommendations and safety of retreatment. *Journal of Crohn’s and Colitis*.

[51] Agarwal A., Kedia S., Jain S. (2018). High risk of tuberculosis during infliximab therapy despite tuberculosis screening in inflammatory bowel disease patients in India. *Intestinal Research*.

[52] Jauregui-Amezaga A., Turon F., Ordás I. (2013). Risk of developing tuberculosis under anti-TNF treatment despite latent infection screening. *Journal of Crohn’s and Colitis*.

[53] WHO (2019). *WHO Guidelines on Tuberculosis Infection Prevention and Control*.

